# Beyond Glycemic Control: GLP-1 Receptor Agonists and Their Impact on Calcium Homeostasis in Real-World Patients

**DOI:** 10.3390/jcm13164896

**Published:** 2024-08-19

**Authors:** Bandar T. Alenezi, Nadra Elfezzani, Rukhsana Uddin, Hinali Patel, Sydney Chester, Ahmed Abdelmaksoud, Mohammad H. Hussein, Sawsan A. Zaitone, Manal S. Fawzy, Hani Aiash, Eman A. Toraih

**Affiliations:** 1 Department of Pharmacology, Faculty of Medicine, Northern Border University, Arar 91431, Saudi Arabia; bandar.alenezi@nbu.edu.sa; 2 Tulane School of Public Health and Tropical Medicine, New Orleans, LA 70112, USA; nadra.elfezzani@gmail.com; 3 Women Medical and Dental College, Khyber Medical University Peshawar, Abbottabad 22080, Pakistan; rukhsanauddinmd@gmail.com; 4 School of Medicine, Louisiana State University, New Orleans, LA 70112, USA; hpat12@lsuhsc.edu; 5 School of Medicine, Tulane University, New Orleans, LA 70112, USA; schester1@tulane.edu; 6 Department of Internal Medicine, University of California, Riverside, CA 92521, USA; aabelma@medsch.ucr.edu; 7 Department of Family Medicine, Ochsner Clinic Foundation, New Orleans, LA 70112, USA; mohamed.hussein@ochsner.org; 8 Department of Pharmacology and Toxicology, Faculty of Pharmacy, University of Tabuk, Tabuk 71491, Saudi Arabia; szaitone@ut.edu.sa; 9 Department of Biochemistry, Faculty of Medicine, Northern Border University, Arar 91431, Saudi Arabia; 10 Center for Health Research, Northern Border University, Arar 91431, Saudi Arabia; 11 SUNY Upstate Medical University, Syracuse, NY 13210, USA; aiashh@upstate.edu; 12 Department of Surgery, School of Medicine, Tulane University, New Orleans, LA 70112, USA; 13 Medical Genetics Unit, Department of Histology and Cell Biology, Suez Canal University, Ismailia 41522, Egypt

**Keywords:** GLP-1R agonists, type 2 diabetes, obesity, calcium homeostasis, propensity-matched controls, cardiovascular events, all-cause mortality, personalized treatment, long-term safety, healthcare utilization

## Abstract

**Background/Objectives:** The effect of glucagon-like peptide-1 receptor (GLP-1R) agonists on calcium homeostasis is poorly understood. This study aimed to investigate the association between GLP-1R agonist use and the risk of hypocalcemia and/or hypercalcemia, as well as other clinical outcomes. **Methods:** A retrospective cohort study used de-identified patient data from the TriNetX Global Collaborative Network, including 15,655 adult patients prescribed GLP-1R agonists and 15,655 propensity-matched controls. Outcomes included hypocalcemia, hypercalcemia, emergency visits, hospitalizations, cardiovascular events, and all-cause mortality. **Results:** GLP-1R agonist use was associated with a reduced risk of hypocalcemia (2.7% vs. 5.5%, RR 0.49, 95% CI: 0.44–0.55) but an increased risk of hypercalcemia (2.3% vs. 1.1%, RR 2.02, 95% CI: 1.69–2.42). The effect on hypocalcemia was most pronounced during the first six months of treatment. Among individual agents, tirzepatide showed the most pronounced effect, reducing hypocalcemia risk by 63% while increasing hypercalcemia risk by 85%. Semaglutide demonstrated similar effects, while dulaglutide and liraglutide showed modest effects. Furthermore, GLP-1R agonist use was associated with reduced risks of emergency visits (RR 0.57, 95% CI: 0.54–0.60), hospitalizations (RR 0.40, 95% CI: 0.36–0.44), cardiovascular events, and all-cause mortality (HR 0.27, 95% CI: 0.21–0.36). **Conclusions**: GLP-1R agonists exhibit a complex influence on calcium homeostasis, reducing hypocalcemia risk while increasing hypercalcemia risk. Beyond calcium regulation, these medications significantly reduce healthcare utilization, improve cardiovascular outcomes, and decrease mortality. Further research is needed to elucidate the mechanisms behind the differential effects of individual GLP-1R agonists, particularly tirzepatide, to optimize personalized treatment approaches and long-term safety.

## 1. Introduction

The incidence of type 2 diabetes and obesity has reached epidemic proportions, representing a significant public health challenge worldwide [1]. Among the evolving therapeutic options, glucagon-like peptide-1 receptor (GLP-1R) agonists have gained prominence due to their robust efficacy in improving glycemic control and conferring additional physiological benefits [2]. These drugs, including liraglutide, exenatide, and dulaglutide, mimic the effects of the incretin hormone GLP-1, resulting in multiple beneficial actions such as slowed gastric emptying, enhanced glucose-dependent insulin secretion, reduced appetite, decreased inappropriate glucagon secretion, and promotion of beta-cell proliferation [3]. 

Beyond their primary metabolic effects, GLP-1R agonists have been found to influence calcium handling and homeostasis in various tissues. Research has shown that these drugs modulate calcium dynamics in pancreatic β-cells [4], muscle cells, and cardiac myocytes [5]. Moreover, the impact of GLP-1R agonists on bone metabolism is an area of growing interest and concern [6]. Notably, some studies have reported improvements in bone mineral density and reductions in fracture risk among patients using GLP-1R agonists [7,8]. However, there remain significant concerns regarding their potential effects on bone turnover and mineralization, necessitating a thorough understanding of these implications [8,9]. These findings underscore the importance of evaluating the role of GLP-1R agonists in bone metabolism in tandem with their effects on calcium homeostasis for optimal patient care and treatment optimization [10]. 

Calcium homeostasis is critical for numerous physiological processes, including muscle contraction, neurotransmitter release, and intracellular signaling [11]. Maintaining proper calcium balance is essential for overall health, and disruptions in calcium homeostasis can profoundly affect cellular function and metabolism [12]. The multifaceted impact of GLP-1R agonists on calcium homeostasis in real-world settings involves several possible mechanisms. These drugs have been found to enhance insulin secretion, improve insulin sensitivity, and promote weight loss, all of which can indirectly affect calcium metabolism [13]. Additionally, improvements in cardiovascular outcomes associated with GLP-1R agonists may be mediated, in part, by their effects on calcium regulation [14]. However, the precise mechanisms by which GLP-1R agonists influence calcium homeostasis and the translation of these effects into clinical outcomes remain incompletely understood.

Glucagon-like peptide-1 is well recognized for its insulinotropic effects, and evidence suggests it plays a role in bone homeostasis, potentially acting as a mediator within the entero-osseous axis. This hypothesis posits that gut-derived hormones, like GLP-1, may have systemic regulatory roles in bone turnover and mineralization [15]. Despite these intriguing connections, there is a notable paucity of large-scale studies specifically examining the association between GLP-1R agonist use and the risk of hypocalcemia or hypercalcemia [16]. Addressing this gap is fundamental, particularly as maintaining a constant plasma calcium level is crucial for many health processes, including maintaining proper bone mineral density and ensuring effective cellular function [17].

Given the expanding clinical application of GLP-1R agonists for both diabetes management and weight control, clarifying their safety profile concerning electrolyte homeostasis is increasingly important. As patients receive these treatments over extended periods, understanding any potential risks associated with calcium homeostasis becomes essential for optimizing therapeutic outcomes and preventing adverse events.

This study aims to explore, for the first time, whether treatment with GLP-1R agonists is associated with an elevated risk of blood calcium dysregulation and to discern the differential impacts of individual GLP-1R agonists on this parameter. By conducting a retrospective cohort analysis using real-world data from the “TriNetX electronic health record network”, we hope to provide insightful findings that could lead to more personalized and effective treatment strategies for patients with type 2 diabetes and related conditions. Our research intends not only to deepen the understanding of GLP-1R agonists’ role in calcium homeostasis but also to pave the way for future studies investigating their broader implications in metabolic and bone health.

## 2. Materials and Methods

### 2.1. Study Design and Data Source

We conducted a retrospective cohort study using de-identified patient data from the “TriNetX Global Collaborative Network”, a federated health research platform that provides access to electronic health records from over 150 million patients across more than 125 healthcare organizations worldwide. The TriNetX platform allows for real-time access to harmonized clinical data while ensuring patient privacy through de-identification and aggregation of results. Data were accessed on 6 July 2024.

### 2.2. Study Population

The study cohort included adult patients (age ≥ 18 years) with a prescription for a GLP-1R agonist (including liraglutide, semaglutide, dulaglutide, or tirzepatide). The index date was defined as the date of the first GLP-1R agonist prescription. Patients were excluded if they had a history of bariatric surgery, thyroid disorders, or hypocalcemia in the three months prior to the index date. A comparison cohort of patients not prescribed GLP-1R agonists was identified using propensity score matching based on age, sex, race, body mass index, comorbidities (including diabetes, hypertension, and chronic kidney disease), and use of medications that may affect calcium homeostasis. Inclusion and exclusion criteria are listed in Table 1 and Table 2.

The study cohort included adult patients (age ≥ 18 years) with a prescription for a GLP-1R agonist (including liraglutide, semaglutide, dulaglutide, or tirzepatide). The index date was defined as the date of the first GLP-1R agonist prescription. Patients were included if they had a diagnosis of obesity (BMI ≥ 30 kg/m^2^ or ICD-10 code for obesity).

Patients were excluded if they had a history of neoplasms, chronic kidney disease, osteoporosis, inflammatory bowel syndrome, parathyroidectomy, thyroidectomy, blood transfusion, vitamin D deficiency, corticosteroid use, pancreatitis, magnesium deregulation, bariatric surgery, or use of medications known to affect calcium levels (e.g., bisphosphonates, denosumab, cinacalcet, cisplatin, diuretics, proton pump inhibitors, certain antibiotics) in the three months prior to the index date. Patients with prior use of discontinued GLP-1R agonists (lixisenatide, albiglutide, exenatide) were also excluded (detailed inclusion and exclusion criteria are listed in Table 1 and Table 2).

A comparison cohort of patients not prescribed GLP-1R agonists was identified using propensity score matching based on age, sex, race, body mass index, smoking, alcohol use, and comorbidities (including diabetes and hypertension). 

### 2.3. Exposure and Outcome Definitions

The primary exposure was defined as having at least one prescription for a GLP-1R agonist. The primary outcome was incident hypocalcemia, defined as either a serum calcium level ≤ 8.4 mg/dL, ionized calcium ≤ 1.10 mmol/L (or ≤4.60 mg/dL), or an ICD-10 diagnosis code for hypocalcemia (E83.51) occurring after the index date.

Secondary outcomes included serum parathyroid hormone (PTH) and vitamin D levels, emergency department visits, hospitalizations, osteoporosis, tetany/spasms/myalgia, seizures, arrhythmias, heart failure, depression/hallucinations/confusion, and all-cause mortality. These outcomes were identified using laboratory values, procedure codes, and ICD-10 diagnosis codes, as detailed in Supplementary Table S1.

### 2.4. Follow-Up and Time Periods

Patients were followed from the index date until the occurrence of hypocalcemia, death, loss to follow-up, or end of the study period (31 December 2023), whichever came first. We analyzed outcomes over three distinct periods: 0–6 months, 6–12 months, and 12–24 months after the index date to assess both short-term and longer-term risks. Additionally, we conducted an “anytime” analysis for all outcomes, including mortality, to capture events occurring at any point during the entire follow-up period after the index date.

### 2.5. Statistical Analysis

Baseline characteristics were compared between the GLP-1R agonist and control groups using standardized mean differences. The cumulative incidence of hypocalcemia was estimated using Kaplan–Meier methods, and hazard ratios (HRs) with 95% confidence intervals (CIs) were calculated using Cox proportional hazard models. Models were adjusted for potential confounders not accounted for in the propensity score matching. Subgroup analyses were performed to assess the risk of hypocalcemia across different GLP-1R agonists and patient characteristics. All analyses used the TriNetX network’s analytics features, which utilize R statistical software (version 4.3.0). *p*-values < 0.05 were considered statistically significant.

### 2.6. Ethical Considerations

This study used de-identified patient data and was determined to be exempt from full review by our institutional review board. This study followed the Declaration of Helsinki and good clinical practice guidelines.

## 3. Results

### 3.1. Characteristics of the Study Population

Our study analyzed data from 128,886,898 adult patients (≥18 years old) across 125 healthcare organizations. After applying inclusion and exclusion criteria, we identified 15,655 patients in the treated group (GLP-1R agonist users) and 217,365 patients in the control group. Table 3 presents the demographic and clinical characteristics of the study population before and after propensity score matching.

Before matching, the treated group was slightly older than the control group (43.2 ± 13.0 vs. 42.6 ± 15.2 years, *p* < 0.001). Both groups had a higher proportion of females, with a slightly lower percentage in the treated group (55.2% vs. 56.5%, *p* = 0.002). White patients were more prevalent in the treated group (61.4% vs. 57.5%, *p* < 0.001) than controls. Regarding comorbidities, the treated group had significantly higher rates of diabetes (14.3% vs. 4.5%, *p* < 0.001) and hypertension (9.4% vs. 7.5%, *p* < 0.001), but lower rates of nicotine dependence (0.9% vs. 1.6%, *p* < 0.001) and alcohol use disorder (0.2% vs. 0.3%, *p* = 0.004). After propensity score matching, the characteristics between the treated and control groups were well balanced, ensuring that any differences observed in outcomes between the treated and control groups are more likely attributable to the effect of GLP-1R agonist treatment rather than underlying differences in patient characteristics.

### 3.2. Overall GLP-1R Agonist Use

Our analysis of all GLP-1R agonists combined revealed significant differences in outcomes between matched treated and control groups (Table 4). GLP-1R agonist use was associated with a reduced risk of hypocalcemia (2.7% vs. 5.5%, *p* < 0.001; RR 0.49, 95% CI: 0.44–0.55) and improved clinical outcomes, particularly in cardiovascular health. The incidence of congestive heart disease was lower in the treated group (1.1% vs. 2.8%, *p* < 0.001; RR 0.41, 95% CI: 0.34–0.49), as was the incidence of arrhythmia (1.6% vs. 2.8%, *p* < 0.001; RR 0.56, 95% CI: 0.48–0.66).

Healthcare utilization was also favorably impacted, with GLP-1 agonist use associated with fewer emergency visits (12.7% vs. 21.9%, *p* < 0.001; RR 0.57, 95% CI: 0.54–0.60) and hospitalization visits (3.1% vs. 7.7%, *p* < 0.001; RR 0.40, 95% CI: 0.36–0.44). Notably, all-cause mortality was significantly lower in the GLP-1 agonist group (0.4% vs. 1.5%, *p* < 0.001; HR 0.27, 95% CI: 0.21–0.36).

However, the GLP-1 agonist group exhibited an increased risk of hypercalcemia (2.3% vs. 1.1%, RR 2.02, 95% CI: 1.69–2.42, *p* < 0.001) and a slight elevation in neuropsychiatric symptoms, with depression/hallucination/confusion being more common in the treated group (13.5% vs. 11.4%, *p* < 0.001; RR 1.18, 95% CI: 1.11–1.25). 

### 3.3. Individual GLP-1R Agonist Analyses

Separate propensity score matching was conducted for each GLP-1 receptor agonist, resulting in balanced treated and control groups: semaglutide (n = 7349 pairs), liraglutide (n = 1513 pairs), dulaglutide (n = 2695 pairs), and tirzepatide (n = 1379 pairs). This matching process, based on demographics and comorbidities, enabled a more precise evaluation of each drug’s unique effects. Subgroup analyses of these matched pairs showed varying degrees of impact on calcium levels (Table 5).

Tirzepatide demonstrated the most pronounced effect, reducing hypocalcemia risk by 63% (RR 0.37, 95% CI: 0.236–0.563) while increasing hypercalcemia risk by 85% (RR 1.85, 95% CI: 1.079–3.171). Semaglutide showed similar trends, with a 62% reduction in hypocalcemia (RR 0.38, 95% CI: 0.318–0.459) and a 51% increase in hypercalcemia (RR 1.51, 95% CI: 1.178–1.935). Dulaglutide and liraglutide exhibited modest effects. Dulaglutide reduced hypocalcemia risk by 34% (RR 0.66, 95% CI: 0.53–0.84, *p* = 0.001) and increased hypercalcemia risk by 76% (RR 1.76, 95% CI: 1.24–2.51, *p* = 0.001). Liraglutide showed a 40% reduction in hypocalcemia risk (RR 0.60, 95% CI: 0.43–0.82, *p* = 0.001), but the increase in hypercalcemia risk was not statistically significant (RR 1.47, 95% CI: 0.79–2.71, *p* = 0.21).

**Figure 1:**
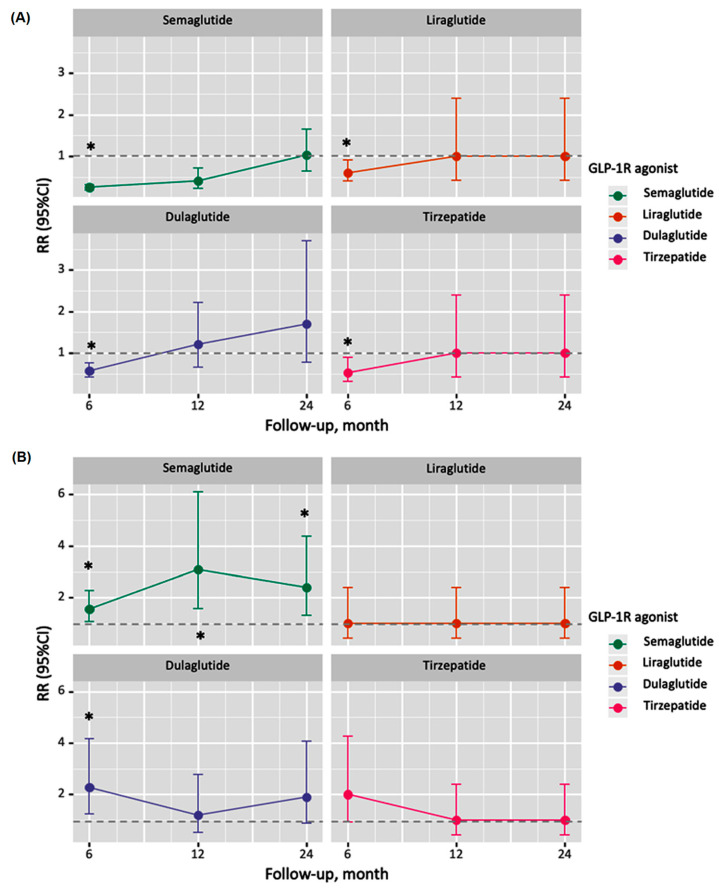
Time-dependent analysis of hypocalcemia and hypercalcemia risks associated with GLP-1R agonist use. (**A**) Risk ratios for hypocalcemia at 0–6 months, 7–12 months, and 13–24 months after treatment initiation for each GLP-1R agonist. (**B**) Risk ratios for hypercalcemia at 0–6 months, 7–12 months, and 13–24 months after treatment initiation for each GLP-1R agonist. Error bars represent 95% confidence intervals. The dotted line (i.e., the control) indicates a risk ratio of 1 (no effect). Values below 1 indicate reduced risk, while above 1 indicate increased risk. Semaglutide (blue), liraglutide (red), dulaglutide (green), and tirzepatide (purple) are represented for each time interval. Asterisks (*) denote statistically significant differences (*p* < 0.05) compared to the control group.

The time-dependent analysis demonstrated that the impact on hypocalcemia was most pronounced in the first six months of treatment for all drugs, with diminishing effects observed at 12 and 24 months (Figure 1A and Table S2). In contrast, the risk of hypercalcemia tended to persist or increase over time, particularly for semaglutide and dulaglutide (Figure 1B and Table S2).

A detailed time-series analysis of various outcomes for individual GLP-1R agonists compared to their propensity-matched controls is provided in Table 6. All drugs significantly reduced emergency visits and hospitalizations in the first six months, with semaglutide and tirzepatide maintaining this effect over time. For cardiovascular outcomes, such as congestive heart failure and arrhythmia, protective effects were shown for all drugs in the first six months, with semaglutide maintaining this effect at 24 months. Apart from tirzepatide, there is an increased risk of depression, hallucination, or confusion, especially at 12 and 24 months for most drugs.

## 4. Discussion

This large-scale retrospective cohort study provides compelling evidence for the complex effects of GLP-1R agonists on calcium homeostasis and clinical outcomes in a real-world setting. Our findings reveal a paradoxical impact on calcium levels, with these drugs significantly reducing the risk of hypocalcemia and increasing the risk of hypercalcemia. This “calcium conundrum” presents a fascinating aspect of GLP-1R agonist therapy that warrants further investigation.

The observed 51% reduction in hypocalcemia risk across all GLP-1R agonists is a novel finding with important clinical implications. This protective effect was consistent across individual agents, with tirzepatide showing the most pronounced reduction (63%). The mechanism underlying this protection against hypocalcemia is not fully understood but may be related to the known effects of GLP-1R agonists on bone metabolism and calcium homeostasis. GLP-1R agonists promote bone formation while inhibiting bone resorption [6]. However, the concurrent doubling of hypercalcemia risk (102% increase) highlights the complex interplay between these agents and calcium regulation. With an increased hypercalcemia risk, individuals are more prone to experiencing complications such as nephrolithiasis, distal renal tubular acidosis, cholelithiasis, and some mood disorders [18].

The basis for direct skeletal effects, and therefore indirect influence of calcium homeostasis, by GLP-1R agonists remains unclear. Pre-clinical studies have demonstrated that GLP-1 plays a role in regulating skeletal homeostasis, suggesting that GLP-1 may promote bone formation [7,19], though clinical studies have not supported these effects [20,21,22]. The role and related mechanisms of GLP-1R agonists in bone metabolism as they relate to calcium homeostasis need to be further elucidated and clarified [23,24]. 

Interestingly, our time-dependent analysis revealed that the effects on hypocalcemia were most pronounced in the first six months of treatment, with diminishing effects observed at 12 and 24 months. In contrast, the risk of hypercalcemia tended to persist or increase over time. This temporal pattern suggests a dynamic influence of GLP-1 receptor agonists on calcium metabolism that evolves throughout treatment. It raises crucial questions about the underlying mechanisms and emphasizes the need for careful monitoring of calcium levels in patients on these medications, especially in the early months of treatment and with newer agents like tirzepatide.

The differential effects observed among individual GLP-1R agonists are noteworthy. Tirzepatide and semaglutide demonstrated the most pronounced effects on hypocalcemia and hypercalcemia, while dulaglutide and liraglutide showed modest impacts. These differences may be attributed to the unique pharmacological properties of each agent, particularly tirzepatide’s dual action on gastric inhibitory polypeptide (GIP) and GLP-1 receptors [25]. GIP receptors have been found in osteoblasts and osteoclasts; they have been shown to protect osteoblasts from apoptosis and inhibit bone resorption [26]. Further research is needed to elucidate the specific mechanisms underlying the differential effects of individual GLP-1R agonists and their clinical significance [27].

The time-dependent analysis demonstrated that the impact on hypocalcemia was most pronounced in the first six months of treatment for all drugs, with diminishing effects observed at 12 and 24 months (Figure 1A). In contrast, the risk of hypercalcemia tended to persist or increase over time, particularly for semaglutide and dulaglutide (Figure 1B). Beyond calcium homeostasis, our study corroborates and extends previous findings on the cardiovascular benefits of GLP-1R agonists. We observed significant reductions in emergency visits, hospitalizations, cardiovascular events, and all-cause mortality. The 73% reduction in all-cause mortality is particularly striking and suggests that the benefits of these drugs may be even more pronounced in real-world settings than in controlled clinical trials. Further supporting the cardioprotective role of the GLP-1R agonists, evidence from other studies suggests that the agonists assist in promoting myocardial glucose uptake while reducing oxidative stress and decreasing apoptosis [28]. Additionally, the literature explains the role of GLP-1R agonists in increasing coronary blood flow through inducing vasodilation [29]. These vasodilatory effects also reduce systemic vascular resistance, thus lowering blood pressure [30]. Furthermore, the indirect effect of GLP-1R agonists in reducing cardiovascular morbidity via decreasing blood glucose levels, postprandial lipemia, inflammation, blood pressure, and body weight was reported by Ussher and Drucker in their interesting review [31].

The slight increase in reports of depression, hallucinations, or confusion in the GLP-1R agonist group is an unexpected finding that requires further investigation [32]. It is unclear whether this represents an adverse effect or is related to other factors, such as increased healthcare utilization or improved survival in this group [33].

Our study has several strengths, including its large sample size, real-world setting, and comprehensive assessment of multiple outcomes. However, it also has limitations inherent to retrospective studies using electronic health records. These include potential confounding by indication, lack of randomization, and possible incomplete capture of all relevant clinical information. Additionally, the relatively short follow-up period for newer agents like tirzepatide limits our ability to draw conclusions about their long-term effects.

## 5. Conclusions

This study provides robust evidence for the complex effects of GLP-1R agonists on calcium homeostasis and clinical outcomes in real-world patients. We found that these agents reduce hypocalcemia risk but increase hypercalcemia risk, with effects varying among individual drugs and over time. Significant improvements in cardiovascular outcomes and reduced all-cause mortality were also observed. These findings have important clinical implications, suggesting the need for close monitoring of calcium levels, especially during the first six months of treatment, and consideration of individual agent characteristics when selecting therapy. This study highlights the importance of using GLP-1R agonists not only for their effects on glycemic control and weight loss but also for their impact on calcium balance and cardiovascular health. Future research should focus on prospective studies to confirm these findings, explore underlying mechanisms, and assess long-term safety and efficacy, particularly for newer agents like tirzepatide. While GLP-1R agonists offer significant benefits in diabetes and obesity management, their complex effects on calcium homeostasis necessitate careful patient monitoring and individualized treatment approaches.

## Figures and Tables

**Table 1:** Inclusion criteria for the study participants.

Criteria	Category	Code	Description
Obesity	Diagnosis	UMLS:ICD10CM:Z68.3	Body mass index [BMI] 30–39, adult
	Diagnosis	UMLS:ICD10CM:Z68.4	Body mass index [BMI] 40 or greater, adult
	Diagnosis	UMLS:ICD10CM:E66.0	Obesity due to excess calories (at least 18 years old at event)
GP1R agonist	Medication	NLM:RXNORM:2601723	Tirzepatide
	Medication	NLM:RXNORM:1991302	Semaglutide
	Medication	NLM:RXNORM:475968	Liraglutide
	Medication	NLM:RXNORM:1551291	Dulaglutide

**Table 2:** Exclusion criteria for the study participants.

Criteria	Category	Code	Description
Neoplasm	Diagnosis	UMLS:ICD10CM:C00-D49	Neoplasms
Chronic kidney disease	Diagnosis	UMLS:ICD10CM:N18	Chronic kidney disease (CKD)
	Diagnosis	UMLS:ICD10CM:N18.6	End-stage renal disease
Osteoporosis	Diagnosis	UMLS:ICD10CM:M81	Osteoporosis without current pathological fracture
	Diagnosis	UMLS:ICD10CM:M80	Osteoporosis with current pathological fracture
Inflammatory bowel syndrome	Diagnosis	UMLS:ICD10CM:K50	Crohn’s disease [regional enteritis]
	Diagnosis	UMLS:ICD10CM:K51	Ulcerative colitis
	Diagnosis	UMLS:ICD10CM:K52.8	Other specified noninfective gastroenteritis and colitis
	Diagnosis	UMLS:ICD10CM:K50-K52	Noninfective enteritis and colitis
Parathyroidectomy	Procedure	UMLS:SNOMED:53304009	Parathyroidectomy
	Procedure	UMLS:CPT:60500	Parathyroidectomy or exploration of parathyroid(s)
	Procedure	UMLS:ICD10PCS:0GBR0ZZ	Excision of parathyroid gland, open approach
	Procedure	UMLS:ICD10PCS:0GBR4ZZ	Excision of parathyroid gland, percutaneous endoscopic approach
	Procedure	UMLS:ICD10PCS:0G5R0ZZ	Destruction of parathyroid gland, open approach
Thyroidectomy	Procedure	UMLS:SNOMED:13619001	Thyroidectomy
	Procedure	UMLS:CPT:60240	Thyroidectomy, total or complete
	Procedure	UMLS:CPT:1009039	Thyroidectomy, total or subtotal for malignancy
	Procedure	UMLS:CPT:60252	Thyroidectomy, total or subtotal for malignancy; with limited neck dissection
	Procedure	UMLS:CPT:60260	Thyroidectomy, removal of all remaining thyroid tissue following previous removal of a portion of thyroid
	Procedure	UMLS:CPT:1009043	Thyroidectomy, including substernal thyroid
	Procedure	UMLS:CPT:60271	Thyroidectomy, including substernal thyroid; cervical approach
	Procedure	UMLS:CPT:60254	Thyroidectomy, total or subtotal for malignancy; with radical neck dissection
	Procedure	UMLS:CPT:60270	Thyroidectomy, including substernal thyroid; sternal split or transthoracic approach
	Procedure	UMLS:SNOMED:359882009	Thyroidectomy with laryngectomy
	Procedure	UMLS:SNOMED:24443003	Total thyroidectomy
	Procedure	UMLS:SNOMED:30956003	Subtotal thyroidectomy
	Procedure	UMLS:SNOMED:52826006	Substernal thyroidectomy
	Procedure	UMLS:SNOMED:24711004	Partial substernal thyroidectomy
	Procedure	UMLS:SNOMED:237486002	Total thyroidectomy with cervical lymph node dissection
	Procedure	UMLS:SNOMED:712978001	Revision thyroidectomy
	Procedure	UMLS:SNOMED:359884005	Radical laryngopharyngectomy with synchronous thyroidectomy
	Procedure	UMLS:ICD9CM:06.5	Substernal thyroidectomy
	Procedure	UMLS:SNOMED:719753008	Completion thyroidectomy
	Procedure	UMLS:SNOMED:767574004	Operation on parathyroid gland during thyroidectomy
Transfusion	Procedure	UMLS:SNOMED:301842006	Intravenous blood transfusion
Vitamin D deficiency	Diagnosis	UMLS:ICD10CM:E55	Vitamin D deficiency
Corticosteroids	Medication	NLM:ATC:H02A	Corticosteroids for systemic use, plain
	Medication	NLM:ATC:R01AD	Corticosteroids
	Medication	NLM:ATC:C05AA	Corticosteroids
	Medication	NLM:ATC:S02B	Corticosteroids
	Medication	NLM:ATC:S03B	Corticosteroids
	Medication	NLM:VA:HS050	Adrenal corticosteroids
Pancreatitis	Diagnosis	UMLS:ICD10CM:K86.1	Other chronic pancreatitis
Magnesium deregulation	Diagnosis	UMLS:ICD10CM:E83.42	Hypomagnesemia
	Diagnosis	UMLS:ICD10CM:E83.41	Hypermagnesemia
Discontinued GLP1R agonist	Medication	NLM:RXNORM:1440051	Lixisenatide
	Medication	NLM:RXNORM:1534763	Albiglutide
	Medication	NLM:RXNORM:60548	Exenatide
Bariatric surgery	Diagnosis	UMLS:ICD10CM:Z98.84	Bariatric surgery status
	Procedure	UMLS:CPT:1007385	Bariatric Surgery Procedures
	Procedure	UMLS:CPT:1007392	Other Procedures on the Stomach
Medications affecting calcium level	Medication	NLM:RXNORM:9384	Rifampin
	Medication	NLM:RXNORM:8183	Phenytoin
	Procedure	UMLS:CPT:1011174	Phenytoin
	Medication	NLM:RXNORM:8134	Phenobarbital
	Procedure	UMLS:CPT:80184	Phenobarbital
	Medication	NLM:RXNORM:46041	Alendronate
	Medication	NLM:RXNORM:115264	Ibandronate
	Procedure	UMLS:HCPCS:J1740	Injection, ibandronate sodium, 1 mg
	Medication	NLM:RXNORM:73056	Risedronate
	Medication	NLM:RXNORM:77655	Zoledronic acid
	Procedure	UMLS:HCPCS:J3489	Injection, zoledronic acid, 1 mg
	Medication	NLM:RXNORM:993449	Denosumab
	Medication	NLM:RXNORM:407990	Cinacalcet
	Medication	NLM:RXNORM:2555	Cisplatin
	Medication	NLM:ATC:C03	Diuretics
	Medication	NLM:VA:CV700	Diuretics
	Medication	NLM:RXNORM:4603	Furosemide
	Medication	NLM:RXNORM:38413	Torsemide
	Procedure	UMLS:HCPCS:J3265	Injection, torsemide, 10 mg/mL
	Medication	NLM:RXNORM:7646	Omeprazole
	Medication	NLM:RXNORM:283742	Esomeprazole
	Medication	NLM:RXNORM:10627	Tobramycin
	Medication	NLM:RXNORM:1596450	Gentamicin

**Table 3:** Characteristics of the study population.

Characteristics	Before Matching		*p*-Value	After Matching		*p*-Value
	Treated	Control		Treated	Control	
Count	15,655	217,365		15,655	15,655	
Age at Index	43.2 ± 13.0	42.6 ± 15.2	**<0.001**	43.2 ± 13.0	43.2 ± 13.0	0.98
Sex						
Female	8640 (55.2%)	122,706 (56.5%)	**0.002**	8640 (55.2%)	8641 (55.2%)	0.99
Male	5702 (36.4%)	82,179 (37.8%)		5702 (36.4%)	5702 (36.4%)	
Race						
White	9612 (61.4%)	125,055 (57.5%)	**<0.001**	9612 (61.4%)	9612 (61.4%)	0.95
Black	2689 (17.2%)	39,580 (18.2%)		2689 (17.2%)	2693 (17.2%)	
Asian	334 (2.1%)	3466 (1.6%)		334 (2.1%)	331 (2.1%)	
Ethnicity						
Not Hispanic/Latino	9207 (58.8%)	118,365 (54.5%)	**<0.001**	9207 (58.8%)	9208 (58.8%)	0.99
Hispanic or Latino	1487 (9.5%)	30,914 (14.2%)	**<0.001**	1487 (9.5%)	1483 (9.5%)	0.94
Comorbidities						
Smoking	147 (0.9%)	3508 (1.6%)	**<0.001**	147 (0.9%)	130 (0.8%)	0.30
Alcohol use disorder	27 (0.2%)	736 (0.3%)	**0.004**	27 (0.2%)	19 (0.1%)	0.23
Diabetes	2249 (14.3%)	9739 (4.5%)	**<0.001**	2249 (14.3%)	2222 (14.1%)	0.66
Hypertension	1477 (9.4%)	16,401 (7.5%)	**<0.001**	1477 (9.4%)	1444 (9.2%)	0.52

Data are presented as numbers (percentages) or mean ± standard deviation. Two-sided Chi-square or Student’s *t*-tests were used. Bold values indicate significance at *p*-values less than 0.05.

**Table 4:** Comparison of outcomes between GLP-1R agonist users and controls.

Outcome	Treated	Control	*p*-Value	RR (95%CI)
**Calcium homeostasis**				
Hypocalcemia	426 (2.7%)	860 (5.5%)	**<0.001**	0.49 (0.44, 0.55)
Hypercalcemia	355 (2.3%)	175 (1.1%)	**<0.001**	2.02 (1.69, 2.42)
Serum calcium	9.41 ± 0.44	9.27 ± 0.54	**<0.001**	---
Serum PTH	57.97 ± 32.97	56.24 ± 35.11	0.67	---
Serum vitamin D	29.07 ± 13.53	26.2 ± 13.24	**<0.001**	---
**Healthcare utilization**				
Emergency visit	1981 (12.7%)	3430 (21.9%)	**<0.001**	0.57 (0.54, 0.60)
Inpatient hospitalization	483 (3.1%)	1203 (7.7%)	**<0.001**	0.40 (0.36, 0.44)
**Clinical outcomes**				
Osteoporosis	10 (0.1%)	10 (0.1%)	1.0	1.0 (0.41, 2.40)
Tetany/spasm/myalgia	280 (1.8%)	360 (2.3%)	**0.001**	0.77 (0.66, 0.90)
Seizures	85 (0.5%)	162 (1%)	**<0.001**	0.52 (0.40, 0.68)
Congestive heart disease	179 (1.1%)	433 (2.8%)	**<0.001**	0.41 (0.34, 0.49)
Arrhythmia	250 (1.6%)	441 (2.8%)	**<0.001**	0.56 (0.48, 0.66)
Depression/hallucination/confusion	2110 (13.5%)	1788 (11.4%)	**<0.001**	1.18 (1.11, 1.25)
**Case-fatality rate**				
All-cause mortality	68 (0.4%)	230 (1.5%)	**<0.001**	0.27 (0.21, 0.36) *

Data are presented as n (%) for categorical variables and mean ± standard deviation for continuous variables. RR = relative risk; CI = confidence interval. *p*-values are derived from chi-square tests for categorical variables and *t*-tests for continuous variables. RR and 95% CI are not applicable (---) for continuous variables. * Hazard ratio (HR) is reported. Bold values indicate significance at *p*-values less than 0.05.

**Table 5:** Comparison of hypocalcemia and hypercalcemia risks among individual GLP-1R agonists.

Outcome	Treated	Control	*p*-Value	RR (95%CI)
**Hypocalcemia**				
Semaglutide	152 (2.1%)	398 (5.4%)	**<0.001**	0.38 (0.31, 0.45)
Liraglutide	59 (3.9%)	98 (6.5%)	**0.001**	0.60 (0.43, 0.82)
Dulaglutide	113 (4.2%)	169 (6.3%)	**0.001**	0.66 (0.53, 0.84)
Tirzepatide	27 (2%)	74 (5.4%)	**<0.001**	0.37 (0.23, 0.56)
**Hypercalcemia**				
Semaglutide	154 (2.1%)	102 (1.4%)	**0.001**	1.51 (1.17, 1.93)
Liraglutide	25 (1.7%)	17 (1.1%)	0.21	1.47 (0.79, 2.71)
Dulaglutide	83 (3.1%)	47 (1.7%)	**0.001**	1.76 (1.24, 2.51)
Tirzepatide	37 (2.7%)	20 (1.5%)	**0.023**	1.85 (1.07, 3.17)

Data are presented as n (%) for categorical variables. RR = relative risk; CI = confidence interval. *p*-values are derived from chi-square tests. Bold values indicate significance at *p*-values less than 0.05.

**Table 6:** Time-series analysis for outcomes for individual drugs of propensity-matched treated cohort and controls.

Outcome	Time Window	Semaglutide	Liraglutide	Dulaglutide	Tirzepatide
Hypocalcemia	6 months	**0.25 (0.2–0.33)**	**0.6 (0.4–0.91)**	**0.57 (0.42–0.76)**	**0.53 (0.31–0.89)**
	12 months	**0.4 (0.22–0.71)**	1.0 (0.42–2.4)	1.21 (0.66–2.22)	1.0 (0.42–2.4)
	24 months	1.03 (0.64–1.66)	1.0 (0.42–2.4)	1.7 (0.78–3.71)	1.0 (0.42–2.4)
Hypercalcemia	6 months	**1.56 (1.06–2.28)**	1.0 (0.42–2.4)	**2.27 (1.24–4.15)**	2.0 (0.94–4.26)
	12 months	**3.09 (1.57–6.1)**	1.0 (0.42–2.4)	1.2 (0.52–2.77)	1.0 (0.42–2.4)
	24 months	**2.4 (1.32–4.38)**	1.0 (0.42–2.4)	1.9 (0.89–4.08)	1.0 (0.42–2.4)
Emergency visit	6 months	**0.38 (0.34–0.43)**	**0.53 (0.42–0.66)**	**0.67 (0.57–0.78)**	**0.35 (0.26–0.46)**
	12 months	**0.74 (0.62–0.87)**	0.75 (0.53–1.06)	0.98 (0.76–1.27)	**0.6 (0.39–0.93)**
	24 months	**0.57 (0.48–0.68)**	**0.69 (0.49–0.96)**	0.96 (0.75–1.23)	**0.54 (0.36–0.81)**
Inpatient hospitalization	6 months	**0.27 (0.23–0.33)**	**0.5 (0.36–0.68)**	**0.56 (0.45–0.7)**	**0.24 (0.15–0.39)**
	12 months	**0.53 (0.29–0.98)**	1.0 (0.42–2.4)	1.2 (0.52–2.77)	1.0 (0.42–2.4)
	24 months	**0.54 (0.32–0.91)**	1.2 (0.52–2.77)	1.17 (0.54–2.52)	1.0 (0.42–2.4)
Tetany, spasm, or myalgia	6 months	**0.65 (0.48–0.87)**	**0.36 (0.17–0.73)**	**0.56 (0.34–0.93)**	0.53 (0.25–1.13)
	12 months	0.9 (0.54–1.5)	1.0 (0.42–2.4)	1.08 (0.5–2.37)	1.0 (0.42–2.4)
	24 months	1.08 (0.63–1.86)	1.0 (0.42–2.4)	1.46 (0.72–2.95)	1.0 (0.42–2.4)
Seizures	6 months	**0.44 (0.28–0.7)**	1.0 (0.42–2.4)	0.68 (0.34–1.38)	0.91 (0.39–2.13)
	12 months	0.73 (0.34–1.6)	NA	1.0 (0.42–2.4)	1.0 (0.42–2.4)
	24 months	0.79 (0.4–1.55)	NA	1.0 (0.42–2.4)	1.0 (0.42–2.4)
Congestive heart disease	6 months	**0.3 (0.22–0.42)**	0.71 (0.4–1.26)	**0.37 (0.25–0.56)**	**0.3 (0.15–0.61)**
	12 months	0.93 (0.56–1.56)	1.0 (0.42–2.4)	0.94 (0.48–1.86)	1.0 (0.42–2.4)
	24 months	**0.43 (0.23–0.78)**	1.0 (0.42–2.4)	0.84 (0.43–1.63)	NA
Arrhythmia	6 months	**0.41 (0.31–0.55)**	**0.47 (0.28–0.79)**	**0.26 (0.16–0.44)**	**0.41 (0.23–0.73)**
	12 months	0.57 (0.31–1.06)	1.0 (0.42–2.4)	0.67 (0.3–1.48)	1.0 (0.42–2.4)
	24 months	**0.53 (0.31–0.9)**	1.0 (0.42–2.4)	0.58 (0.28–1.21)	1.0 (0.42–2.4)
Depression, hallucination, or confusion	6 months	1.06 (0.95–1.17)	1.05 (0.84–1.3)	**1.32 (1.11–1.57)**	1.09 (0.86–1.37)
	12 months	**1.75 (1.46–2.09)**	**1.51 (1.06–2.15)**	**1.89 (1.38–2.61)**	1.45 (0.99–2.14)
	24 months	**1.47 (1.21–1.78)**	**1.57 (1.04–2.39)**	**1.88 (1.34–2.64)**	1.25 (0.79–1.98)

Data are reported as relative risk (RR) and 95% confidence interval (CI). Bold values indicate significance *p*-values less than 0.05.

## Data Availability

Restrictions apply to the availability of these data. Data were obtained from the TriNetX database and are available at https://trinetx.com/ with the permission of the TriNetX database authority.

## Supplementary Materials

The following supporting information can be downloaded at https://www.mdpi.com/article/10.3390/jcm13164896/s1, Table S1: Outcome measures; Table S2: Overall serum levels and time points of the studied parameters in the study groups.
