# Supplementary material for: Beyond Glycemic Control: GLP-1 Receptor Agonists and Their Impact on Calcium Homeostasis in Real-World Patients
Source: J Clin Med. 2024 Aug 19;13(16):4896. doi: 10.3390/jcm13164896 (PMC11355112; doi:10.3390/jcm13164896)
Supplement: Supplementary file 1 [file jcm-13-04896-s001.zip › jcm-3133780-supplementary.pdf]

Table S1. Outcome measures.

Criteria	Category	Code	Description
Hypocalcemia	Laboratory	TNX:9022	Calcium [Mass/volume] in Serum, Plasma or Blood (at most 8.40 mg/dL (most recent occurrence))
	Laboratory	UMLS:LNC:38250-7	Calcium [Mass/volume] in Specimen (at most 8.40 mg/dL (most recent occurrence))
	Laboratory	UMLS:LNC:1994-3	Calcium ionized [Moles/volume] in Blood (at most 1.10 mmol/L (most recent occurrence))
	Laboratory	TNX:LG655-7	Calcium ionized [Moles/volume] in Serum, Plasma or Blood (at most 1.10 mmol/L (most recent occurrence))
	Laboratory	UMLS:LNC:1995-0	Calcium ionized [Moles/volume] in Serum or Plasma (at most 1.10 mmol/L (most recent occurrence))
	Laboratory	UMLS:LNC:59471-3	Calcium ionized [Mass/volume] in Venous blood (at most 4.60 mg/dL (most recent occurrence))
Serum Calcium	Laboratory	TNX:9022	Calcium [Mass/volume] in Serum, Plasma or Blood (most recent occurrence)
Serum PTH	Laboratory	UMLS:LNC:2731-8	Parathyrin. intact [Mass/volume] in Serum or Plasma (most recent occurrence)
Serum Vitamin D	Laboratory	TNX:9034	Calcidiol [Mass/volume] in Serum or Plasma (most recent occurrence)
Emergency visit	Visit	UMLS:HL7V3.0:VisitType:EMER	Visit: Emergency
	Procedure	UMLS:CPT:1013711	Emergency Department Services
	Procedure	UMLS:CPT:99284	Emergency department visit for the evaluation and management of a patient, which requires a medically appropriate history and/or examination and moderate level of medical decision making

	Procedure	UMLS:CPT:99283	Emergency department visit for the evaluation and management of a patient, which requires a medically appropriate history and/or examination and low level of medical decision making
	Procedure	UMLS:CPT:99285	Emergency department visit for the evaluation and management of a patient, which requires a medically appropriate history and/or examination and high level of medical decision making
	Procedure	UMLS:CPT:99282	Emergency department visit for the evaluation and management of a patient, which requires a medically appropriate history and/or examination and straightforward medical decision making
	Procedure	UMLS:SNOMED:50849002	Emergency room admission
	Procedure	UMLS:SNOMED:182813001	Emergency treatment
	Procedure	UMLS:SNOMED:183452005	Emergency hospital admission
Hospitalization visit	Procedure	UMLS:CPT:1013659	Hospital Inpatient and Observation Care Services
	Procedure	UMLS:CPT:1013682	Hospital Inpatient or Observation Discharge Services
	Procedure	UMLS:CPT:1013683	Hospital inpatient or observation discharge day management
	Procedure	UMLS:CPT:99239	Hospital inpatient or observation discharge day management; more than 30 minutes on the date of the encounter
	Procedure	UMLS:CPT:99238	Hospital inpatient or observation discharge day management; 30 minutes or less on the date of the encounter
	Procedure	UMLS:CPT:1013648	Hospital Observation Services
	Procedure	UMLS:SNOMED:32485007	Hospital admission

Osteoporosis	Diagnosis	UMLS:ICD10CM:M81	Osteoporosis without current pathological fracture
	Diagnosis	UMLS:ICD10CM:M80	Osteoporosis with current pathological fracture
	Diagnosis	UMLS:ICD10CM:M81.8	Other osteoporosis without current pathological fracture
	Diagnosis	UMLS:ICD10CM:M80.8	Other osteoporosis with current pathological fracture
Tetany, spasm, or myalgia	Diagnosis	UMLS:ICD10CM:R29.0	Tetany
	Diagnosis	UMLS:ICD10CM:R25.2	Cramp and spasm
	Diagnosis	UMLS:ICD10CM:M79.1	Myalgia
Seizures	Diagnosis	UMLS:ICD10CM:R56	Convulsions, not elsewhere classified
Arrhythmia	Diagnosis	UMLS:ICD10CM:I49	Other cardiac arrhythmias
	Diagnosis	UMLS:ICD10CM:I49.9	Cardiac arrhythmia, unspecified
	Diagnosis	UMLS:ICD10CM:I49.8	Other specified cardiac arrhythmias
Congestive heart disease	Diagnosis	UMLS:ICD10CM:I50	Heart failure
Depression, hallucination, confusion	Diagnosis	UMLS:ICD10CM:F32	Depressive episode
	Diagnosis	UMLS:ICD10CM:F32.A	Depression, unspecified
	Diagnosis	UMLS:ICD10CM:R44.3	Hallucinations, unspecified
	Diagnosis	UMLS:ICD10CM:R41.0	Disorientation, unspecified
All-cause mortality	Demographics	Deceased	Deceased
	Diagnosis	UMLS:ICD10CM:R99	Ill-defined and unknown cause of mortality

Table S2. Overall serum levels and time points of the studied parameters in the study groups.

	6 months			12 months			24 months		
	Treated	Control	*p*-value	Treated	Control	*p*-value	Treated	Control	*p*-value
Serum Ca	9.4 ± 0.5	9.2 ± 0.6	**<0.001**	9.43 ± 0.5	9.34 ± 0.6	**<0.001**	9.42 ± 0.4	9.36 ± 0.5	**<0.001**
Serum PTH	55.63 ± 30.6	54.37 ± 33.6	0.83	64.54 ± 41	52.33 ± 30.1	0.32	52.71 ± 31.5	35.14 ± 16.3	0.10
Serum Vitamin D	29.36 ± 13.1	24.96 ± 12.7	**<0.001**	29.78 ± 13.4	31.36 ± 18.8	0.52	30.36 ± 13.9	32.71 ± 23.3	0.34

Data are reported as mean ± standard deviation. Bold values indicate significance at a *p*-value less than 0.05.
